# Functional In Vitro Assessment of rAAV-Delivered Retinol Dehydrogenase 12 (RDH12) Activity

**DOI:** 10.3390/ijms27031366

**Published:** 2026-01-29

**Authors:** Polina Pavlova, Marina Averina, Dzerassa Gurtsieva, Alima Galieva, Roman A. Ivanov, Alexander Karabelsky, Ekaterina Minskaia

**Affiliations:** Translational Medicine Research Center, Sirius University of Science and Technology, 1 Olympic Avenue, 354340 Sochi, Russia

**Keywords:** retinopathy, Leber’s congenital amaurosis, LCA, inherited retinal disorder, IRD, AAV, gene replacement therapy

## Abstract

Gene replacement therapy can be used for the treatment of hereditary retinopathies, such as retinol dehydrogenase 12 (RDH12)-associated Leber congenital amaurosis 13 (LCA13); however, the lack of animal models accurately mimicking the human disease phenotype requires the initial in vitro confirmation of therapy efficacy. Two synthetic serotypes (2.7m8 and PHP.S) of adeno-associated virus (AAV) were tested against the natural serotypes (5 and 9) with the aim of increasing the transduction efficiency and delivery of the green fluorescent protein (GFP) in HEK293 and ARPE-19 cells. The three most efficient serotypes were then used for the delivery of RDH12, followed by the assessment of its functional activity in the transduced cells. In the in vitro test system, a cassette encoding GFP and the wild-type (wt) RDH12 was delivered into ARPE-19 and HEK293 cells by rAAV 5, PHP.S, and 7m8 at 30K and 60K VG/cell. RDH12 mutants pThr155Ile (RDH12mut) and Met1* (RDH12sc) were used to mimic the RDH12-associated pathology. Transduction efficiency and protein expression were assessed by flow cytometry, fluorescence microscopy, and Western blotting. Percentages of AAV7m8-transduced GFP+ cells 1.5- and 6.4-times higher were observed as compared to AAV5 and AAV.PHP.S, respectively. 4-hydroxynonenal (4-HNE), more toxic to the cells with dysfunctional RDH12, was used on cells expressing the three RDH12wt versions. Following treatment with 100 μM 4-HNE, 2.6 (AAV5) and 8.8 (AAV7m8) times more cells co-expressing RDH12wt and GFP were alive as compared to the cells expressing only GFP. The number of live RDH12wt-expressing cells was also 32 and 9.6 times higher than that of RDH12sc-expressing cells and the negative control (NC), respectively. The developed approach enables the functional assessment of RDH12 replacement therapy only in rAAV-transduced cells and demonstrates that rAAV7m8 is the most efficient serotype for this purpose.

## 1. Introduction

Leber’s congenital amaurosis (LCA) is a group of inherited autosomal recessive or autosomal dominant retinal diseases with early severe manifestation, which is characterized by phenotypic and genotypic heterogeneity [1]. So far, twenty-nine genes associated with diverse LCA phenotypes have been described [2]. Mutations in these genes cause dysfunction or complete loss of the affected proteins, which ultimately leads to the death of rod and cone photoreceptors. Clinically significant genetic variants of these genes are responsible for approximately 70–80% of LCA cases, and new genes associated with this pathology are still being discovered. The pathological mechanisms can generally be subdivided into those related to phototransduction (GUCY2D, AIPL1, RD3, and KCNJ13), retinoid cycle (RPE65, LRAT, and RDH12), ciliary transport (LCA5, CEP290, RPGRIP1, SPATA7, TULP1, and IQCB1), photoreceptor morphogenesis (CRX, CRB1, GDF6, and PRPH2), guanine synthesis (IMPDH1), and photoreceptor differentiation (OTX2) [3].

LCA accounts for approximately 5% of all hereditary retinopathies, occurs with a frequency of 1/81,000 to 1/30,000 [4,5], and up to 10% of these belong to LCA type 13 [6], associated with mutations in the *RDH12* gene, which encodes the enzyme retinol dehydrogenase 12 (RDH12). Like most other LCA types, LCA13 is an autosomal recessive disease associated with visual cycle disorders. RDH12 participates in retinoid metabolism, which is critically important for the normal function of retinal photoreceptors. The lack of enzyme’s functional activity leads to disruption of the photoreceptor chromophore repair processes and contributes to the accumulation of toxic metabolites, which causes damage and subsequent death of photosensitive retinal cells and leads to premature vision loss [7].

In addition, mutations in *RDH12* leading to a decrease or loss of its expression can cause the accumulation of lipid peroxidation products, such as the medium-chain aldehydes trans-2-nonenal, 4-hydroxynonenal (4-HNE), and cis-6-nonenal, and to a lesser extent by-products of the retinoid cycle, in particular A2E [8,9]. 4-HNE by-products are substrates for RDH12 and converted by it into biologically safe metabolites. However, in the case of LCA13, 4-HNE by-products spontaneously react with cysteine, histidine, and lysine residues in cellular proteins, as well as with glutathione, further causing oxidative stress and eventually leading retinal cells to apoptosis due to their increased sensitivity to oxidative damage when exposed to light. Observations of the clinical picture describe two possible scenarios for disease development: 1. rapid and early degeneration of the pericentral retina and 2. slowly progressive degeneration of the peripheral retina, which may link such phenotypes with the main (visual cycle) and auxiliary (antioxidant protection) functions of RDH12 [10].

Advances in molecular medicine over the past decade have significantly expanded the possibilities of diagnosis, monitoring, and treatment of patients with inherited retinal diseases (IRD). New treatment approaches include stem cell-based therapy [11], optogenetic approaches based on the introduction of photosensitive channel proteins (opsins) into functional, initially non-photosensitive retinal cells [12], the use of RNA interference to correct defective splicing [13], genomic editing using the CRISPR/Cas system and base and prime editing technologies [14], and viral vector-mediated gene replacement therapy [15]. The latter is the most promising approach and is based on the adeno-associated virus (AAV)-mediated delivery of a healthy gene copy into cells. The relative simplicity of AAV production, the possibility of specific tissue-targeted delivery due to the pronounced tropism of various AAV serotypes, stable transgene expression, and relatively low immunogenicity, which additionally can be reduced by intravitreal or subretinal injections, together make the AAV platform very attractive for such therapeutic purposes [16].

Over the past 8 years, the Food and Drug Administration (FDA) approved Luxturna (Voretigene neparvovec) for the treatment of IRD [17]. Several more therapeutic candidates from MeiraGTx aimed at the treatment of various inherited retinal dystrophies (LCA1 with a mutation in *GUCY2D*, LCA4 with a mutation in *AIPL1*, and LCA13 with a mutation in *RDH12*) have received the rare childhood disease drug status and are at the clinical trial or accelerated review stage by the FDA [18,19].

Based on the demonstrated success of the AAV-mediated gene replacement therapy for other disorders, this platform also promises to demonstrate clinical efficacy for LCA13. However, the development of any therapy requires the mandatory preclinical stages aimed at in vitro and in vivo assessment of the safety and efficacy. Unfortunately, *RDH12* knockout models do not accurately reflect the phenotypic picture of LCA13, thus limiting functional studies [20]. Retinal degeneration is not observed in laboratory mice with RDH12^−/−^ knockout due to differences in the metabolism of retinoid cycle products [21]. The only animal model that may reproduce the LCA13 phenotype after *RDH12* knockout is Danio rerio [22]. In addition to in vivo stages, preclinical in vitro modeling also has great potential, but most studies (mainly based on induced pluripotent stem cells) are in the early stages of development [23,24]. Therefore, it is essential to confirm the efficiency of gene replacement therapy at the stage of in vitro work and preclinical tests [25]. At present, in vivo studies allow the confirmation of RDH12 activity based on retinoid cycle metabolism [21]. This approach of evaluating alternative substrates is labor-intensive and expensive, and a simple, straightforward test that allows the assessment of RDH12 activity has so far remained elusive.

In this study, two ultimate goals were pursued: 1. selection of the most efficient rAAV serotype for the transduction of both human embryonic kidney (HEK293) and the more clinically relevant retinal pigment epithelium (ARPE-19) cells, and 2. development of a functional test for the assessment of the AAV-delivered RDH12 activity in the transduced cells.

## 2. Results

### 2.1. Assessment of rAAV5, rAAV9, rAAV2.7m8, and rAAV.PHP.S Tropism in HEK293 and ARPE-19 Cell Lines

**Figure 1:**
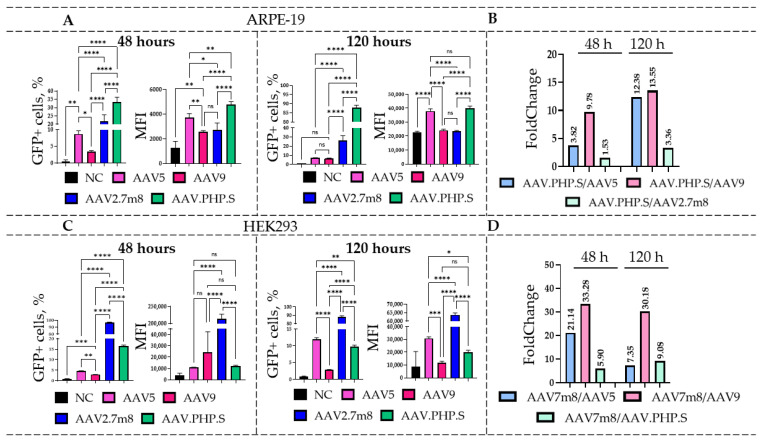
Transduction efficiency of the four AAV serotypes delivering GFP. Flow cytometry analysis of the rAAV5, rAAV9, rAAV2.7m8, and rAAV.PHP.S-transduced ARPE-19 (**A**) and HEK293 (**C**) cells was performed at 48 and 120 h post-transduction. The fold change differences between the most efficient serotypes, rAAV.PHP.S (ARPE-19 cells) and rAAV2.7m8 (HEK293 cells), and other serotypes are shown in panels (**B**,**D**), respectively. One-factor analysis of variance (ANOVA) revealed statistically significant differences (*p*-value < 0.0001) between all serotypes with the exception of rAAV9 and rAAV5 after transduction of ARPE-19 cells, while a high level of significance was demonstrated between all AAV serotypes in HEK293 cells. (*) *p*-value < 0.05, (**) *p*-value < 0.01, (***) *p*-value < 0.001, (****) *p*-value < 0.0001, insignificant (ns) *p*-value > 0.05.

Transduction of HEK293 and ARPE-19 cell lines with natural (rAAV5 and rAAV9) and synthetic (rAAV2.7m8 and rAAV.PHP.S) AAV serotypes, delivering GFP, was performed at a dose of 6 × 10^4^ VG/cell (the viral titer, estimated by qPCR, was in the range of 1 × 10^10^–1 × 10^12^ VG/L). rAAV5, rAAV9, and rAAV2.7m8 serotypes were chosen for their previously confirmed tropism for retinal pigment epithelial cells [26], while the synthetic rAAV.PHP.S, developed by directed evolution of rAAV9 for the transduction of the peripheral nervous system cells, was expected to efficiently transduce retinal cells [27]. The flow cytometry analysis of HEK293 and ARPE-19 cells was performed at 48 and 120 h post-AAV transduction (Figure 1).

For the ARPE-19 cell line (Figure 1A), statistically significant differences were observed at 48 h post-transduction for both the percentages of GFP-positive (GFP+) cells and the mean fluorescence intensity (MFI) values (Table S1). rAAV.PHP.S demonstrated the highest transduction efficiency at 48 h with an average of 33.3% GFP+ cells, outperforming the natural serotypes by 9.8 (for rAAV9) and 3.8 (for rAAV5) times (Figure 1B). Transduction by rAAV.PHP.S-GFP also resulted in the highest (4793) MFI values among the four serotypes. Compared to rAAV.PHP.S, only 21.8% of the rAAV2.7m8-transduced cells were GFP+, which was 1.53 times lower ((****) *p*-value < 0.0001). Interestingly, despite the significant increases in the number of GFP+ cells after rAAV2.7m8 transduction compared to the natural serotypes (2.5- and 6.4-fold differences as compared to AAV5 and AAV9, respectively), the MFI values for cells transduced with rAAV5 were 1.4 times higher than those transduced with rAAV2.7m8 ((*) *p*-value < 0.05). A similar overall trend was observed at 120 h with 87.7% GFP+ rAAV.PHP.S-transduced cells, which was 12–13 times higher as compared to the natural serotypes and 3.4 times higher as compared to rAAV2.7m8 (Figure 1B). Statistically significant differences in MFI values were observed for all serotypes with the exception of rAAV9. Interestingly, the novel serotype rAAV.PHP.S, which had not been used previously for these purposes, demonstrated efficient transduction of the ARPE-19 cell line.

The HEK293 cell line was most efficiently transduced by rAAV2.7m8, which was in accordance with our previously published studies [26]. At 48 h (Figure 1C), the synthetic serotypes rAAV2.7m8 and rAAV.PHP.S outperformed the natural serotypes rAAV5 and rAAV9 in both the percentage of GFP+ cells and MFI values. rAAV2.7m8 transduced approximately 96.6% of cells, which was 21.1 and 33.3 times higher as compared to rAAV5 and rAAV9, respectively (Figure 1D). Comparison of the two most effective serotypes, rAAV2.7m8 and rAAV.PHP.S, revealed statistically significant differences ((****) *p*-value < 0.0001) for both the percentages of GFP+ cells and MFI values. The percentage of rAAV2.7m8-transduced GFP+ cells was 5.9 and 21.1 times higher as compared to rAAV.PHP.S and rAAV5, respectively. However, at 120 h post-transduction, the described trend differed considerably. Despite the low percentage of GFP+ rAAV5-transduced cells at 48 h, the number of GFP-expressing cells increased significantly by the 120 h time point as compared to rAAV.PHP.S- ((**) *p*-value < 0.01) and rAAV9-transduced ((****) *p*-value < 0.0001) cells. Nevertheless, rAAV2.7m8 remained the most effective serotype, with 88% and 97% GFP+ cells at both time points. Overall, the number of AAV2.7m8-transduced GFP+ cells was 7.3, 9.1, and 30.2 times higher than that of rAAV5-, rAAV.PHP.S-, and rAAV9-transduced cells. These differences were statistically significant ((****) *p*-value < 0.0001).

### 2.2. Evaluation of the Transduction Efficiency of rAAV5-, rAAV2.7m8-, and rAAV.PHP.S Delivering GFP-T2A-RDH12wt

**Figure 2:**
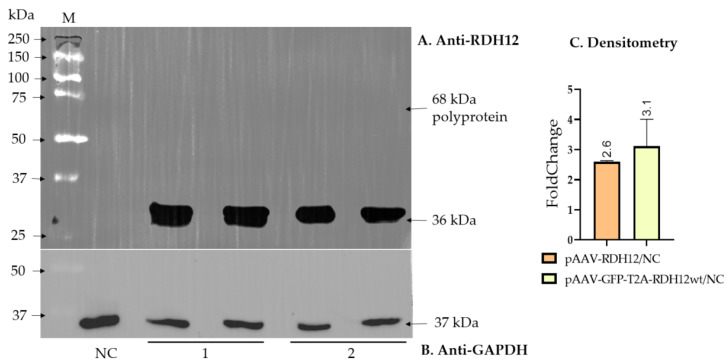
Confirmation of RDH12 expression after transfection of ARPE-19 cells with plasmids pAAV-RDH12 and pAAV-GFP-T2A-RDH12wt. (**A**) Western blotting with anti-RDH12 antibody; (**B**) membrane staining with anti-GAPDH antibody; (**C**) Densitometry analysis of Western blotting data.

To be able to assess the effect of RDH12 replacement therapy on cells that did receive it, we produced three of the most efficient serotypes (rAAV5, rAAV2.7m8, and rAAV.PHP.S) co-expressing RDH12wt and GFP via the self-cleaving T2A peptide (GFP-T2A-RDH12wt). As the ribosome skipping between glycine and proline of the 2A peptide leads to the retention of the 2A-coding sequence of about 25 amino acids at the C-terminus of the first protein while proline remains at the N-terminus of the second protein, and the RDH12 functional domain is located at the C-terminus, the RDH12-encoding sequence was placed after T2A due to the possible loss of the enzyme’s activity. The efficiency of T2A cleavage and the absence of an uncleaved polyprotein were confirmed by Western blotting using anti-RDH12 antibodies (Figure 2A,B).

Densitometry analysis of the Western blotting data further supported comparable RDH12 expression levels from pAAV-GFP-T2A-RDH12wt and pAAV-RDH12 plasmids after transfection when normalized to the housekeeping GAPDH protein.

**Figure 3:**
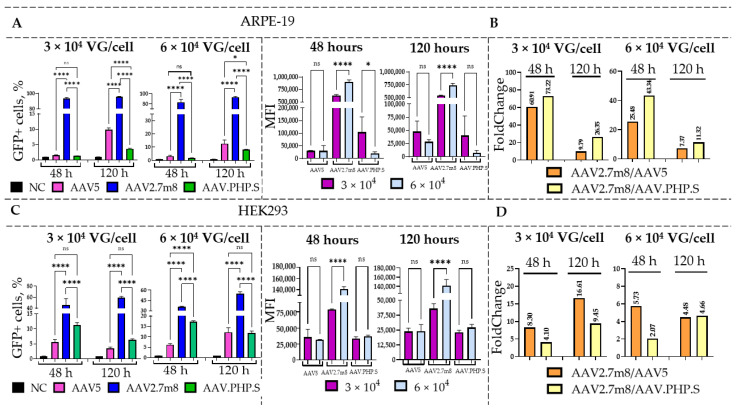
Transduction efficiency of rAAV5, rAAV2.7m8, and rAAV.PHP.S delivering GFP-T2A-RDH12wt. Flow cytometry analysis of ARPE-19 (**A**) and HEK293 (**C**) cells transduced with rAAV2.7m8, rAAV5, and rAAV.PHP.S at two doses (3 × 10^4^ and 6 × 10^4^ VG/cell) was performed at 48 and 120 h post-transduction. Fold change differences between the most efficient serotype, rAAV2.7m8, and two other serotypes in ARPE-19 and HEK293 cells are shown in panels (**B**,**D**), respectively. Statistically significant differences (*p*-value < 0.0001) were observed between rAAV2.7m8 and the other serotypes in all cases; however, differences between rAAV5 and rAAV.PHP.S were only observed at 120 h post-transduction at both viral doses in ARPE-19 cells and at 6 × 10^4^ VG/cell in HEK293 cells. A clear dose-dependent effect based on MFI values was observed only for the rAAV2.7m8 serotype. One-factor analysis of variance (ANOVA) was used for statistical analysis: (*) *p*-value < 0.05, (****) *p*-value < 0.0001, insignificant (ns) *p*-value > 0.05.

To compare the transduction efficiency of the three serotypes, HEK293 and ARPE-19 cells were transduced with rAAV5, rAAV2.7m8, and rAAV.PHP.S viruses delivering GFP-T2A-RDH12wt at the dose of 3 and 6 × 10^4^ VG/cell (Figure 3). At 48 and 120 h post-transduction, the flow cytometry analysis was used to assess the percentage of GFP+ cells and their MFI values. As previously demonstrated, the synthetic serotypes rAAV.PHP.S and rAAV2.7m8 more efficiently transduced both HEK293 and ARPE-19 cells as compared to the natural rAAV5.

As expected, a dose-dependent effect was observed in both cell lines, with a higher percentage of GFP+ cells detected after transduction with a higher viral dose of 6 × 10^4^ as compared to 3 × 10^4^ VG/cell (Table S2). Overall, rAAV2.7m8 demonstrated the highest transduction efficiency in ARPE-19 cells, with the percentages of GFP+ cells ranging from 84 to 95% across both time points and doses. Moreover, it showed a statistically significant increase in GFP+ cells as compared to other serotypes (Figure 3A)**.** Additionally, a significant increase in the MFI values was observed between viral doses 3 × 10^4^ and 6 × 10^4^ VG/cell (increased by 274,750 and 198,000 at 48 and 120 h, respectively), demonstrating a dose-dependent effect. While at 48 h no statistically significant differences were observed between rAAV5 and rAAV.PHP.S ((ns)– *p*-value > 0.05), there was a significant increase in the percentage of GFP+ rAAV5-transduced cells ((*)-*p*-value < 0.05) at 120 h. According to the obtained data, at 48 h post-transduction, 73 and 60 times more rAAV2.7m8-transduced ARPE-19 cells were GFP+ as compared to rAAV.PHP.S- and rAAV5-transduced cells, respectively, when virus was used at 3 × 10^4^ VG/cell. The differences were also observed at 6 × 10^4^ VG/cell at 120 h, with rAAV2.7m8 being 11 times as efficient as rAAV.PHP.S and 7 times as efficient as rAAV5 (Figure 3B). An interesting observation was made in terms of the effect of the transgene size on transduction efficiency of certain AAV serotypes. As demonstrated previously, rAAV.PHP.S was the most efficient serotype for GFP delivery to ARPE-19 cells; however, it was outperformed by rAAV2.7m8 for the delivery of a larger gene of interest (GFP-T2A-RDH12wt) in both cell lines. As the packaging capacity of various AAV serotypes is expected to be identical [28], this is a very interesting and important observation, which may impact future research. At the same time, transduction of HEK293 cells with the rAAV2.7m8 serotype resulted in 35–60% GFP+ cells for both doses and time points (Figure 3C). At 120 h post-transduction with rAAV2.7m8, used at 6 × 10^4^ VG/cell, 4.7- and 4.5-times higher numbers of GFP+ cells were observed as compared to rAAV.PHP.S and rAAV5, respectively (Figure 3D)**.**

### 2.3. Functional Activity of RDH12 Delivered by Three rAAV Serotypes into ARPE-19 Cells

**Figure 4:**
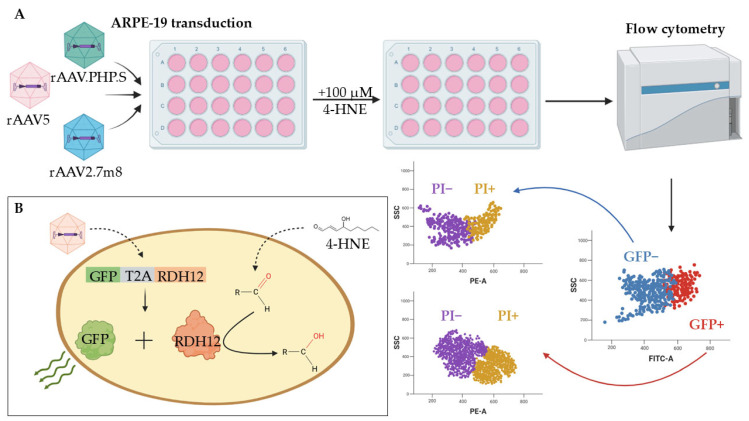
Schematic presentation of the RDH12 functional activity test. The ability of ARPE-19 cells, expressing the rAAV-delivered healthy copy of RDH12, to withstand the 4-HNE cytotoxic effect is assessed by the number of dying/dead (PI+) cells in the GFP+ population transduced with rAAV-GFP-T2A-RDH12 as compared to the untransduced GFP− cells (**A**). The co-expression of RDH12 and GFP allows the identification (and gating on) of the cells that received the therapeutic gene by the GFP fluorescence and the analysis of their viability upon treatment with 4-HNE (**B**).

The RDH12 dysfunction leads to the buildup of toxic metabolites, which causes damage and death of retinal cells. To assess the functional activity of RDH12, the enzyme’s ability to protect the cells from the toxic effects of alternative substrate 4-HNE was tested as shown in Figure 4 (a schematic presentation of the test’s concept). A 100 µM dose of 4-HNE was selected experimentally by assessing the substrate’s cytotoxic effects on ARPE-19 cell morphology and nuclei size (Figure S2).

**Figure 5:**
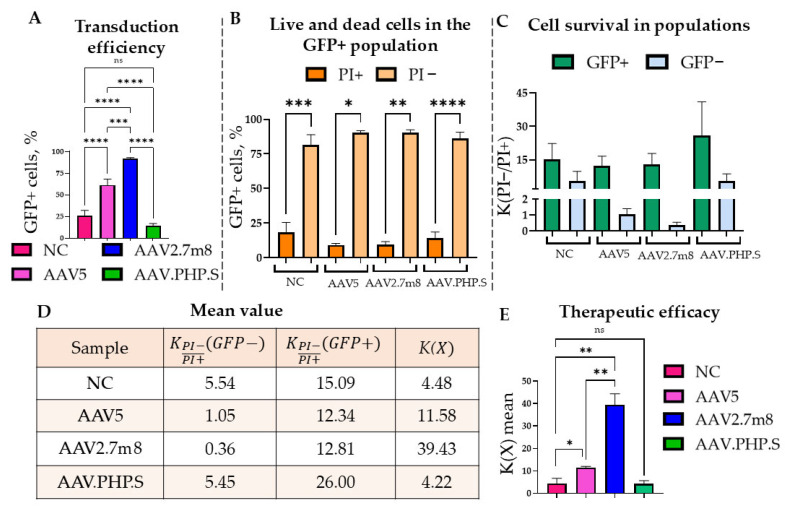
Increased survival of ARPE-19 cells post-RDH12 delivery by the three AAV serotypes under toxic conditions induced by 4-HNE. Flow cytometry analysis was performed at 120 h post-transduction to assess the efficiency of the rAAV5, rAAV2.7m8, and rAAV.PHP.S transduction of ARPE-19 cells (**A**). The percentage of live (PI−) and dying/dead (PI+) cells was assessed in the GFP+ population expressing GFP-T2A-RDH12wt (**B**). The formula for calculating the survival rate (1) indicated that the survival rate for all populations exceeded mean 2, except for the rAAV2.7m8 and rAAV5 serotypes in the GFP+ population (**C**). Table of average coefficients of survival rates, including the overall survival coefficient of the population (**D**). The survival coefficients *K*(*X*), the formula of which is presented in (2), were calculated, and statistically significant differences compared to the NC were observed for rAAV2.7m8 (*p*-value < 0.01) and rAAV5 (*p*-value < 0.05), while no significant differences were found for rAAV.PHP.S (**E**). Unpaired *t*-test analysis of variance: (*) *p*-value < 0.05, (**) *p*-value < 0.01, (***) *p*-value < 0.001, (****) *p*-value < 0.0001, insignificant (ns) *p*-value > 0.05.

Ninety-six hours post transduction with rAAV2.7m8, rAAV5, and rAAV.PHP.S delivering GFP-T2A-RDH12wt and used at 6 × 10^4^ VG/cell, the ARPE-19 cells were treated with 100 µM of 4-HNE and analyzed 24 h later using flow cytometry (Figure 4A). The co-expression of RDH12wt with GFP allowed for the gating on fluorescent cells that also expressed functional RDH12wt and those that did not (Figure 4B). The cell viability was assessed both by the location on the FSC/SSC plot and by the addition of intercalating dye propidium iodide (PI) that penetrates the dying/dead cells (Figure 5 and Figure S3). The rAAV2.7m8-GFP that delivers only GFP was used as a positive control for viral transduction and NC for the RDH12 functional activity.

The most effective rAAV2.7m8 in terms of the percentage of GFP+ cells was 1.5 and 6.4 times more effective than rAAV5 and rAAV.PHP.S, respectively (Figure 5A). rAAVs rarely, if ever, transduce 100% of cells. Therefore, in order to correctly compare the efficiency of the RDH12-mediated cell protection against 4-HNE, it was important to assess the viability of cells that were (GFP+) and were not (GFP−) transduced by the rAAV yet underwent the identical experimental conditions: cells from the same sample (Figure 5B). Thus, the survival rate (i.e., the ratio of PI− to PI+ subpopulations) within one (GFP+ or GFP−) subpopulation was calculated using the Formula (1):

(1) $$K_{\frac{PI-}{PI+}}(X)=\frac{PI-}{PI+},$$

PI− —PI− subpopulation;

PI+—PI+ subpopulation;

$K_{\frac{PI-}{PI+}}(X)$—the ratio of cell survival;

X − GFP+ or GFP− subpopulation on the sample.

The data obtained using formula *K*(*X*) of the total population coefficient (i.e., the ratio of cell survival within the GFP+ to the GFP− populations) was used to compare the cell populations transduced by the different rAAV serotypes and was calculated using the Formula (2):

(2) $$K(X)=\frac{K_{\frac{PI-}{PI+}}\left(GFP+\right)}{K_{\frac{PI-}{PI+}}\left(GFP-\right)},$$

$K_{\frac{PI-}{PI+}}\left(GFP+\right)\;$—the ratio of cell survival within the GFP+;

$K_{\frac{PI-}{PI+}}\left(GFP-\right)$—the ratio of cell survival within the GFP−;

*K*(X)—the total population coefficient

*X*—sample.

Significant statistical differences were observed between the percentages of PI− (live) and PI+ (dying/dead) cells. On average, the percentage of PI+ cells in the GFP+ population was as follows: rAAV5—9.17%, rAAV2.7m8—9.64%, rAAV.PHP.S—13.95%, and the NC—21.4% (Figure 5B and Table S3). The survival rates of the GFP− cells that did not receive therapy did not exceed 2 for the rAAV5 and rAAV2.7m8 and remained at the level of the NC (4.5) for rAAV.PHP.S (5.5) (Figure 5C,D). The described trend was confirmed in all biological repeats, with rAAV7m8 being most effective with a survival rate of 39.4 (Figure 5E). These results were statistically significantly different for the different rAAV serotypes and compared to the NC. Thus, the *K*(*X*) coefficient for rAAV2.7m8 was 8.8 times statistically significantly higher ((**) *p*-value < 0.0021) than that of the NC and 3.4 times higher than that of rAAV5, while rAAV5 was also 2.6 times higher than the control and also statistically significantly different ((*) *p*-value < 0.0332). It is noteworthy that despite the identical design of the transgene cassette delivered by the three serotypes, the rAAV.PHP.S demonstrated both a lower expression level and survival rate as compared to the other two serotypes. Moreover, no statistically significant difference was observed between rAAV.PHP.S and the NC. The possible explanation for this observation could be a low efficiency of entry into the cells or heightened recognition of the virus by the cell with the subsequent destruction of the virus, which requires further research. rAAV2.7m8, on the other hand, not only showed a significant increase in cell survival after exposure to 4-HNE but also more effectively transduced the ARPE-19 cells.

### 2.4. Confirmation of the RDH12 Functional Activity in an In Vitro Disease Model

**Figure 6:**
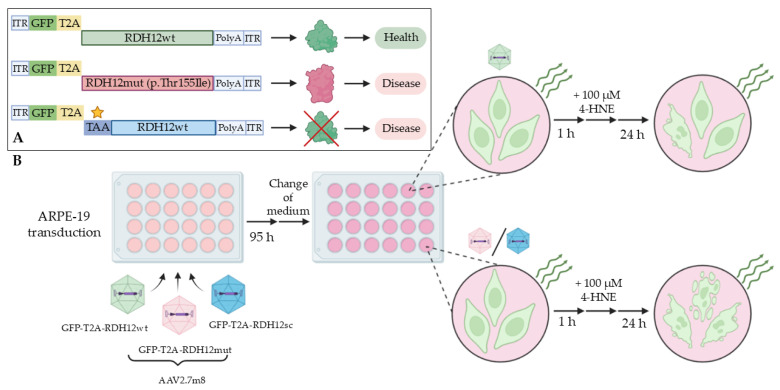
A schematic presentation of the RDH12wt functional activity proof. (**A**) The transgene cassettes coding for RDH12wt, RDH12mut, and RDH12sc, expression of which reflects the enzyme’s function (RDH12wt) or dysfunction (RDH12mut and RDH12sc). The five-pointed star is the location of the stop codon. (**B**) The assessment of the 4-HNE toxic effects on GFP+ ARPE-19 cells expressing RDH12wt, RDH12mut, and RDH12sc.

To additionally prove the protective activity of rAAV-delivered RDH12wt using the developed test system and to confirm the absence of possible non-specific effects (such as viral components, the virus itself, and transgene size), the experimental concept presented in Figure 6 was developed. We produced rAAV2.7m8, the most efficient serotype, delivering two additional mutant versions of RDH12, both mimicking the retinopathy pathology (Figure 6A): 1. RDH12mut with an empirically predicted (but so far experimentally unconfirmed) mutation, Thr155Ile (RDH12mut), and 2. RDH12sc with the complete loss of the protein in the presence of the mRNA transcript (the sequence encoding RDH12 contains a stop codon in the +1 position). In the first scenario (GFP-T2A-RDH12mut), the mutant protein was expected to have reduced functional activity, since this mutation was detected in patients with diagnosed LCA13 [29]. In the second scenario (GFP-T2A-RDH12sc), no RDH12 was expected to be expressed. Upon the 4-HNE treatment (Figure 6B), the ARPE-19 cells expressing GFP-T2A-RDH12wt were expected to have a better ability to survive the toxic effects than the cells expressing either GFP-T2A-RDH12mut or GFP-T2A-RDH12sc.

**Figure 7:**
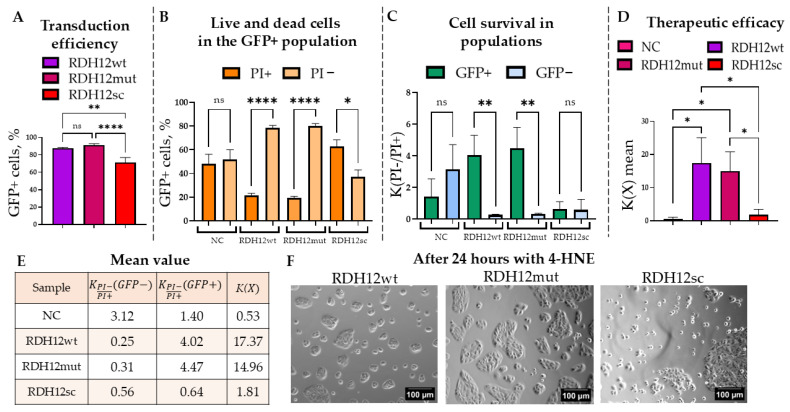
Comparison of the functional activity of RDH12wt, RDH12mut, and RDH12sc expressed in the GFP+ ARPE-19 cells. (**A**). Assessment of transduction efficiency of ARPE-19 cells with rAAV2.7m8 delivering three RDH12 variants was carried out by flow cytometry at 120 h post-transduction. (**B**). GFP+ and PI+ populations were analyzed, along with the ratio of live (PI−) to dead (PI+) cells within both GFP+ and GFP− populations. (**C**). The survival coefficients in these populations were determined, with values for RDH12wt and RDH12mut in the GFP+ population exceeding 4 and for RDH12sc being less than 1. (**D**). The overall survival rates were also calculated for the experimental groups, with the highest values observed for RDH12wt and RDH12mut, which showed statistically significant differences compared to the other samples (*p*-value < 0.0332). (**E**). Table of average coefficients for the survival rates, including the overall survival coefficient of the population. (**F**). Morphological changes in retinal cells 24 h after 4-HNE treatment are shown in the microscopy images. Unpaired *t*-test analysis of variance: (*) *p*-value < 0.05, (**) *p*-value < 0.01, (****) *p*-value < 0.0001, insignificant (ns) *p*-value > 0.05.

Therefore, the ARPE-19 cells were transduced with rAAV2.7m8-GFP-T2A-RDH12wt, rAAV2.7m8-GFP-T2A-RDH12mut, and rAAV2.7m8-GFP-T2A-RDH12sc at 6 × 10^4^ VG/cell. Cells transfected with a plasmid delivering GFP were used both as a positive fluorescence control and a negative RDH12 control. Transduction efficiency was assessed by flow cytometry at 120 h based on the percentage of the GFP+ cells (Figure 7A). In all cases, more than 70% of cells were successfully transduced.

Next, to study the protective effect of the RDH12wt expression post-4-HNE treatment, the percentages of PI− and PI+ cells within the GFP+ population were analyzed (Figure 7B and Table S4). The average percentages of dead GFP+ cells in the cells expressing RDH12wt, RDH12mut, and RDH12sc were 21.44%, 19.20%, and 62.91%, respectively, with statistically significant differences observed between the PI− and PI+ subpopulations in all cases except for the NC. The GFP− population was analyzed similarly, and based on these data. Survival coefficients were calculated for the GFP+ cell populations expressing RDH12 variants and those that did not (GFP−) (Figure 7C). Statistically significant differences were observed between the GFP− and GFP+ populations in samples transduced with either rAAV2.7m8-GFP-T2A-RDH12wt or rAAV2.7m8-GFP-T2A-RDH12mut but not by rAAV2.7m8-GFP-T2A-RDH12sc. The survival coefficient for RDH12sc-expressing cells was less than 1, whereas for RDH12wt and RDH12mut it exceeded 4. The overall cell survival rate for RDH12wt and RDH12mut was 17.37 and 14.96, respectively, which indicates the efficiency of these RDH12 variants in protecting the ARPE-19 cells from the cytotoxic effects of 4-HNE (Figure 7D). Cells that received a healthy RDH12wt gene and RDH12mut survived 32 and 28 times more often than the NC, and 9.6 and 8.3 times more than those that expressed RDH12sc. The average survival coefficients, including the overall population survival coefficients, are presented in the table in Figure 7E. Additionally, the impact of 4-HNE on the morphology of cells transduced with different RDH12 variants can be observed on the microscopy images shown in Figure 7F. Interestingly, while in vitro modeling of the RDH12 loss-of-function was successfully demonstrated upon delivery of the GFP-T2A-RDH12sc, the presence of the mutation in GFP-T2A-RDH12mut did not seem to affect the RDH12 activity in our test system.

## 3. Discussion

Advances in AAV-based gene therapies opened up new possibilities for the treatment of monogenic congenital retinal dystrophies. AAV-mediated gene replacement therapy has a high potential for the treatment of genetic impairments leading to the loss of protein function by restoring protein expression and activity in damaged retinal structures. Although clinical trials primarily use natural AAV serotypes, synthetic variants with improved tropism for specific retinal layers are being actively developed [30]. At the same time, the synthetic AAV2.7m8 already demonstrated great results both in vitro and in mouse and primate in vivo models [31], providing high selectivity and therapeutic potential. The focus of recent studies is shifting towards the safety of vector delivery, enhancing tropism for target cells, dose reduction, and minimization of immune responses [32]. These issues are particularly relevant for new serotypes, given the reported heterogeneity of AAV2.7m8 preparations from different manufacturers, including variability in inflammatory gene expression and cytokine secretion in transduced cells [33].

However, the required prior thorough assessment of the safety and dosage is often achieved at the expense of the experimental data on AAV serotype efficiency in preventing photoreceptor death, especially when model organisms lack a pronounced degenerative phenotype [34]. Thus, for RDH12-associated LCA13, the absence of relevant animal models complicates further research. In addition, it is essential to establish an in vitro model for the future rapid screening of therapeutic molecules.

In the present study, we assessed not only the tropism of the natural (rAAV5 and rAAV9) and engineered (rAAV2.7m8 and rAAV.PHP.S) AAV serotypes in two cell lines (HEK293 and ARPE-19), but also the activity of the rAAV-delivered RDH12wt and its two mutant versions (RDH12 p.Thr155Ile and RDH12sc) in ARPE-19 cells also expressing GFP as a result of a successful viral transduction. Of note, rAAV.PHP.S has not been previously studied as a viral vector for gene replacement therapy for the treatment of retinopathies, yet it proved to be the most effective serotype for the transduction of the ARPE-19 cell line while delivering the short (700 bp) GFP transgene. It showed 1.5–3 times higher transduction efficiency than the rAAV2.7m8 in ARPE-19 cells, while the HEK293 cell line was more efficiently transduced by the latter in accordance with our previously published data [28]. The synthetic serotypes rAAV2.7m8 and rAAV.PHP.S actively transduced HEK293 and ARPE-19 cells as early as 48 h compared to the rAAV5 serotype. However, despite the perceived similar packaging capacity described in the literature [35], a larger transgene cassette had a negative effect on the transduction efficiency of rAAV.PHP.S but not rAAV2.7m8, making rAAV2.7m8 the most promising AAV serotype for ophthalmic research due to its efficient assembly and ability to deliver gene sequences of bigger sizes.

The three most efficient serotypes (rAAV2.7m8, rAAV5, and rAAV.PHP.S) delivering RDH12 together with GFP via the T2A self-cleaving peptide were used as a platform for an in vitro functional test for the evaluation of RDH12 activity post gene replacement therapy. The functional activity of RDH12 was assessed by the restored ARPE-19 cell ability to withstand the cytotoxic effects of 4-HNE [36]. The simultaneous expression of the GFP and the enzyme RDH12 allowed for the detection of the transduced cells and; therefore, gating on the GFP+ cells that also expressed RDH12 and the GFP− cells that did not. To mimic the loss-of-function disease phenotype, we produced rAAVs expressing RDH12mut (c.464C>T, p.Thr155Ile), which was empirically predicted to be pathologic, and RDH12sc with the stop codon at position +1 of RDH12. In the case of the c.464C>T mutant, the threonine in position 155 is replaced by isoleucine near the region presumably responsible for dimer formation [31], therefore, it was expected that the expressed protein would have reduced or no functional activity as a result of its inability to form the active dimer subunits. The introduction of the TAA stop codon into the first position of the RDH12 sequence leads to a complete loss of protein, while the transgene (and the transcribed mRNA) lengths are identical to the RDH12wt cassette. Protein expression was confirmed by Western blotting with anti-RDH12 antibodies and flow cytometry (43.8% of GFP+ cells) after transfection of adhesive HEK293. A complete T2A cleavage efficiency was observed, as no uncleaved polypeptide was detected by Western blotting (Figure S2).

To assess the RDH12 ability to protect the transduced GFP+ cells from the 4-HNE toxic action, the survival rate between the GFP+ and GFP−A cell subpopulations and that of the entire cell population was calculated. The survival rate in the two subpopulations showed a statistically significant difference in all biological repeats for rAAV2.7m8 (the highest efficacy) with a survival rate of 39.4 and rAAV5-11.6. It is interesting to note that the rAAV.PHP.S serotype delivering GFP-T2A-RDH12wt demonstrated a lower transgene expression level and survival, which were comparable to the NC. The reason behind this finding could be the low efficiency of virus penetration into the cells or increased cellular recognition followed by virus destruction, which requires further detailed study of this effect. Interestingly, no expected RDH12mut dysfunction was observed using our experimental design: the survival rate of cells expressing RDH12mut (14.96) was similar to that of cells expressing RDH12wt (17.37). The cells that received either a healthy RDH12wt or RDH12mut copy survived 32 and 28 times more efficiently as compared to the NC, and 9.6 and 8.3 times more efficiently than the cells expressing RDH12sc. Apparently, the presence of the p.Thr155Ile mutation did not have a pronounced pathological effect on the RDH12 activity, unlike the introduction of the stop codon (RDH12sc), leading to the loss of the enzyme’s activity. Taken together, our findings give hope and promise but also demonstrate the limitations of in vitro assays.

For future therapeutic purposes, the delivery of RDH12 will, undoubtedly, be carried out without the reporter protein; however, in present study, special attention was paid to the in vitro model and the assessment of RDH12 activity in the transduced cells that did receive therapy (something that cannot be assessed without the co-expression of the reporter protein). The future study will focus on the delivery of therapeutic gene without GFP and its effect both in vitro and in vivo.

Although the popular HEK293 and the more clinically relevant ARPE-19 cells provide a convenient, efficient platform for both the assessment of rAAV serotypes transduction efficiency and the delivered transgene’s functional activity, in vitro studies do not fully recapitulate the complex architecture of tissue and the immune environment of the human retina. Considering this, future studies may utilize this approach to extend findings on more physiologically relevant models, such as retinal organoids and in vivo systems.

The transition to in vivo research remains a critical step for validating a therapeutic strategy that will ensure the safety, optimal dosage, and translational potential of the developed vector for clinical use. The lack of this data due to the current lack of in vivo models (as described in the Introduction) can be considered a limitation of this study.

## 4. Materials and Methods

### 4.1. Cell Lines

Suspension HEK293 cell lines were cultured in an INFORS HT Multitron incubator shaker (INFORS, Bottmingen, Switzerland) at 37 °C, 80% humidity, 100 rpm, and 5% CO_2_ in Erlenmeyer flasks (Corning, New York, NY, USA) using EmCD HEK293 plus medium (Eminence, Suzhou, China). Adhesive cell lines HEK293 and ARPE-19 were cultured in a BINDER RI 53 CO_2_ incubator (BINDER, Tuttlingen, Germany) at 37 °C and 5% CO_2_ using DMEM HG medium (Pricella, Wuhan, China), supplemented with 5% FBS and 2.5 mM L-glutamine. ARPE-19 cell medium was also supplemented with 1× NEAA.

### 4.2. Construction of Expression Plasmids pAAV-GFP-T2A-RDH12wt, pAAV-GFP-T2A-RDH12mut, and pAAV-GFP-T2A-RDH12sc

The GFP-T2A-RDH12wt plasmid was constructed by overlap extension PCR using pAAV-GFP and pAAV-RDH12wt as templates. The GFP-T2A-RDH12wt sequence was cloned into the pAAV-MCS-CMV vector by restriction digestion to obtain the pAAV- GFP-T2A-RDH12wt plasmid. The introduction of the two mutations was carried out by PCR mutagenesis with overlap extension. The sequence of expression constructs was confirmed by Sanger sequencing (Thermo Fisher Scientific, Waltham, MA, USA), and the size of the obtained plasmids was determined by analytical restriction using *Bam*HI and *Hind*III restriction enzymes (SibEnzyme, Novosibirsk, Russia).

### 4.3. Production of Recombinant Viruses

rAAVs were produced using triple co-transfection with pHelper plasmids (Cell Biolabs, San Diego, CA, USA), pAAV-RC (one of the following: 2/5, 7M8, or PHP.S (Addgene, Watertown, MA, USA), and pAAV-GoI (gene of interest). The GoIs in the pAAV-GoI plasmid were GFP, GFP-T2A-RDH12wt, GFP-T2A-RDH12mut, or GFP-T2A-RDH12sc. Transfection of HEK293 cells was performed 24 h after passage at a concentration of 5 × 10^5^ cells/mL in 300 mL of EmCD HEK293 plus medium. During transfection, a molar plasmid ratio of 2:2:5 (1.5 micrograms of DNA per 1 × 10^6^ viable cells) was used for the three plasmid vectors: pHelper, pAAV-GoI, and pAAV-pRC, while the ratio of pDNA to polyethylenimine (PEI) was kept at 1:4. 120 h after transfection, the cells were lysed by the addition of Tween-20 (Sigma-Aldrich, St. Louis, MO, USA) to a final concentration of 0.05%. After an hour, 30 E.a/mL of benzonase (Diaem, Moscow, Russia) was added to the cells in the presence of 1 mM MgCl_2_ and the incubation continued for an hour. The lysate was clarified by centrifugation at 3000× *g* for 10 min. The supernatant was filtered through a layer of diatomite (Sigma-Aldrich, St. Louis, MO, USA) at 1.5 g per 100 mL of lysate on a vacuum membrane 0.22 µm Stericup PES filter (Merck Millipore, Darmstadt, Germany). The resultant filtrate was concentrated using a Vivaflow 200 HY ultrafiltration system (Sartorius, Göttingen, Germany) and a Masterflex 77921-65 L/S peristaltic pump (Masterflex, Gelsenkirchen, Germany) to a volume of 45 mL. When necessary, the concentrate was additionally filtered through a 0.22-micron syringe PES filter (Jet Biofil, Guangzhou, China). Purification was performed on the affinity sorbent POROS™ CaptureSelect™ AAVX (Thermo Fisher Scientific, Waltham, MA, USA) for rAAV2.7m8 and PHP.S and on the AVB Sepharose High Performance column (Cytiva, Marlborough, MA, USA) for the rAAV5 serotype. The elution buffer for the rAAV5 virus was a solution of 0.5 M arginine, 0.5 M NaCl, 0.1 M citric acid, pH 2.1, and an elution buffer for rAAV2.7m8 and PHP.S included 0.1 M glycine-HCl, 0.05% Tween20, and 0.5 M L-arginine, pH 2.0. The obtained samples were sterilized using a 0.22-micron syringe PES filter (Jet Biofil, Guangzhou, China), stored at −80 °C, and analyzed using qPCR. The titer of the virus, estimated using qPCR, was in the range of 1 × 10^10^–1 × 10^12^ VG/L (viral genomes per liter of culture) [37]. A diagram of the process of production, isolation, and purification of viral particles is shown in Figure S4.

### 4.4. Determination of Virus Titer by Real-Time PCR (qPCR)

qPCR was performed after purification of viral particles to determine the titer of viral genomes. The samples were prepared by DNase I treatment (Sintol, Moscow, Russia) followed by Protease K (Biolabmix, Novosibirsk, Russia). The following reaction mix was used for qPCR: 7.5 µL of 2xHS-High ROX (Biolabmix LLC, Novosibirsk, Russia), 0.5 µL of 10 pcM of F-primer, R-primer ITR probe, and 5 µL of sample after 2 stages of sample preparation in a total volume of 15 µL. A linearized pAAV-GFP plasmid was used as a standard sample, a calibration curve was constructed, and the analysis was performed on a StepOnePlus device (Thermo Fisher Scientific, Waltham, MA, USA) using the Quantification-Standard Curve application of the StepOne SoftWare V2.3 software [26].

### 4.5. Transduction of HEK293 and ARPE-19 Cells

Reverse transduction of cells with serotypes rAAV5, rAAV9, rAAV2.7m8, and rAAV.PHP.S was performed at concentrations of 3 × 10^4^ and 6 × 10^4^ VG/cell with 3–4 repeats. rAAV preparation was added to the suspension of 7.5 × 10^4^ HEK293 and ARPE-19 cells, which were then seeded in a 24-well plate (Jet Biofil, Guangzhou, China). 24 h post-transduction, the medium was replaced. The samples were prepared at 48 and 120 h after transduction. When 4-HNE was used, the medium was changed on day 4 an hour before 4-HNE treatment, and flow cytometry analysis was performed 24 h later (at 120 h post-transduction).

### 4.6. Flow Cytometry

Flow cytometry was performed on a CytoFLEX B2-R2-V0 flow cytofluorimeter (Indianapolis, IN, USA) at 120 h post-transduction with rAAVs or 72 h post-plasmid transfection. Sample preparation was carried out according to the standard protocol with two wash steps in a cooled FACS buffer [38]. To analyze cell viability, the intercalating dye propidium iodide (PI) was added at a dilution of 1:1000. CytExpert software version 1.2 was used for analysis, and gating was performed using FlowJo™ software version 10.10. The analysis was performed using FITC-A parameters to detect a GFP+ population and PE-A to detect PI+ (dead) cells, which also included a late-apoptotic population.

### 4.7. Western Blotting

Electrophoresis in a 10% polyacrylamide gel under denaturing conditions (SDS-PAGE) was performed using the modified Laemmly method. 20 µL of a sample pre-mixed with 7 µL of 4× Laemmly sample buffer (250 mM Tris-HCl pH 6.8, glycerin, 20% 2-mercaptoethanol, 8% sodium dodecyl sulfate (SDS), 0.01% bromophenol blue) was loaded onto the gel. Precision Plus Protein Unstained marker (Bio-Rad, Hercules, CA, USA) was used as a molecular weight marker.

For immunoblotting, an Immobilion-P PVDF membrane (Merck Millipore, Darmstadt, Germany) was pre-activated with 80% ethanol and washed in a transfer buffer. Wet transfer was performed in a Criterion Blotter system (Bio-Rad, Hercules, CA, USA) at a constant current of 35 mA for 16 h at room temperature. The membrane was blocked in a solution of 5% skimmed milk powder in PBST buffer (PBS + 0.5% Tween-20) for 1 h and washed three times with PBST. Next, it was incubated with anti-RDH12 antibodies at a dilution of 1:1000 (Proteintech, Rosemont, IL, USA) in PBST containing 5% skimmed milk for 4 h. After the three wash steps with PBST, the membrane was incubated with secondary antibody (Rabbit IgG Horseradish Peroxidase-conjugated Antibody, R&D Systems, Minneapolis, MN, USA) diluted in PBST (5% skimmed milk) at 1. Following three wash steps in PBST, the proteins were detected using an ECL chemiluminescence detection kit (Thermo Fisher Scientific, Waltham, MA, USA) and the ChemiDoc system (BioRad, Hercules, CA, USA). The same protocol was followed for the detection of GAPDH (Hytest, Moscow, Russia) following the stripping of the membranes.

### 4.8. Statistical Analysis

Statistical analysis was performed using GraphPad Prism 9.3.1 software. Normality was studied for the samples using the Shapiro–Wilk criterion, and one–factor analysis of variance (ANOVA) was used to test the null hypothesis for a normal distribution, in which the level of significant differences are (*) *p*-value < 0.05, (**) *p*-value < 0.01, (***) *p*-value < 0.001, (****) *p*-value < 0.0001, insignificant (ns) *p*-value > 0.05, nonparametric Kruskal–Wallis analysis method was used for abnormal distribution. Unpaired t test analysis of variance: (*) *p*-value< 0.05, (**) *p*-value < 0.01, (***) *p*-value < 0.001, (****) *p*-value < 0.0001, insignificant (ns) *p*-value > 0.05.

## Data Availability

Data available on request.

## Supplementary Materials

The following supporting information can be downloaded at: https://www.mdpi.com/article/10.3390/ijms27031366/s1.

## Abbreviations

The following abbreviations are used in this manuscript:

4-HNE	4-hydroxynonenal
AAV	adeno-associated virus
ANOVA	analysis of variance
ARPE-19	retinal pigment epithelium cells
DNA	deoxyribonucleic acid
ECL	electrochemiluminescence
FDA	Federal Drugs Agency
GFP	green fluorescent protein
GFP+ cell	GFP positive population cells
GFP− cell	GFP negative population cells
GOI	gene of interest
HEK293	human embryonic kidney
IRD	inherited retinal disease
LCA	Leber’s congenital amaurosis
MFI	mean fluorescence intensity
rAAV	adeno-associated viral
RDH12	retinol dehydrogenase 12
RDH12mut	RDH12 with a predicted (but so far experimentally unconfirmed) mutation Thr155Ile
RDH12sc	RDH12 with the stop codon at position +1 relative to RDH12
RDH12wt	healthy RDH12
PCR	polymerase chain reaction
qPCR	quantitative polymerase chain reaction
PEI	polyethylenimine
PI	propidium iodide
PI+ cell	PI-positive population cells
PI− cell	PI-negative population cells
pHelper	helper vector
pRC	plasmid encoding the capsid proteins AAV
VG	vector genome
