# Supplementary material for: Functional In Vitro Assessment of rAAV-Delivered Retinol Dehydrogenase 12 (RDH12) Activity
Source: Int J Mol Sci. 2026 Jan 29;27(3):1366. doi: 10.3390/ijms27031366 (PMC12897934; doi:10.3390/ijms27031366)
Supplement: Supplementary file 1 [file ijms-27-01366-s001.zip › Figure S2.pdf]

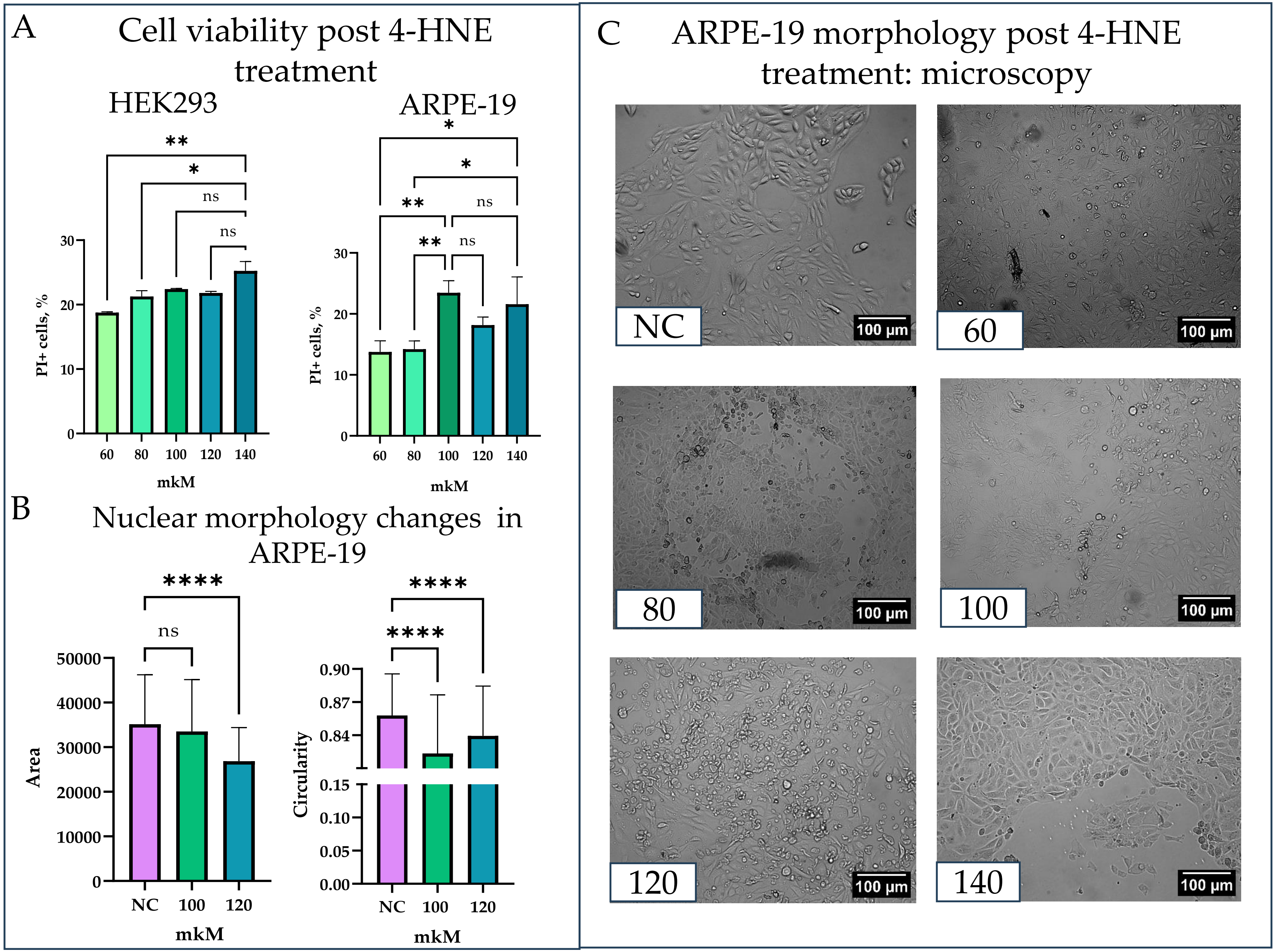

A
Cell viability post 4-HNE treatment
HEK293
ARPE-19
PI+ cells, %
mkM
ns
*
**
B
Nuclear morphology changes in ARPE-19
Area
Circularity
NC
100
120
mkM
ns
****
C
ARPE-19 morphology post 4-HNE treatment: microscopy
NC
60
80
100
120
140
100 μm
