# Supplementary material for: Functional In Vitro Assessment of rAAV-Delivered Retinol Dehydrogenase 12 (RDH12) Activity
Source: Int J Mol Sci. 2026 Jan 29;27(3):1366. doi: 10.3390/ijms27031366 (PMC12897934; doi:10.3390/ijms27031366)

AAV purification

A

Transfection mix

Plasmid	Molar ratio	Total plasmid DNA, µg/million cells	DNA:PEI ratio
pHelper	2	1,5	1:4
GoI	2		
RepCap	5		

B

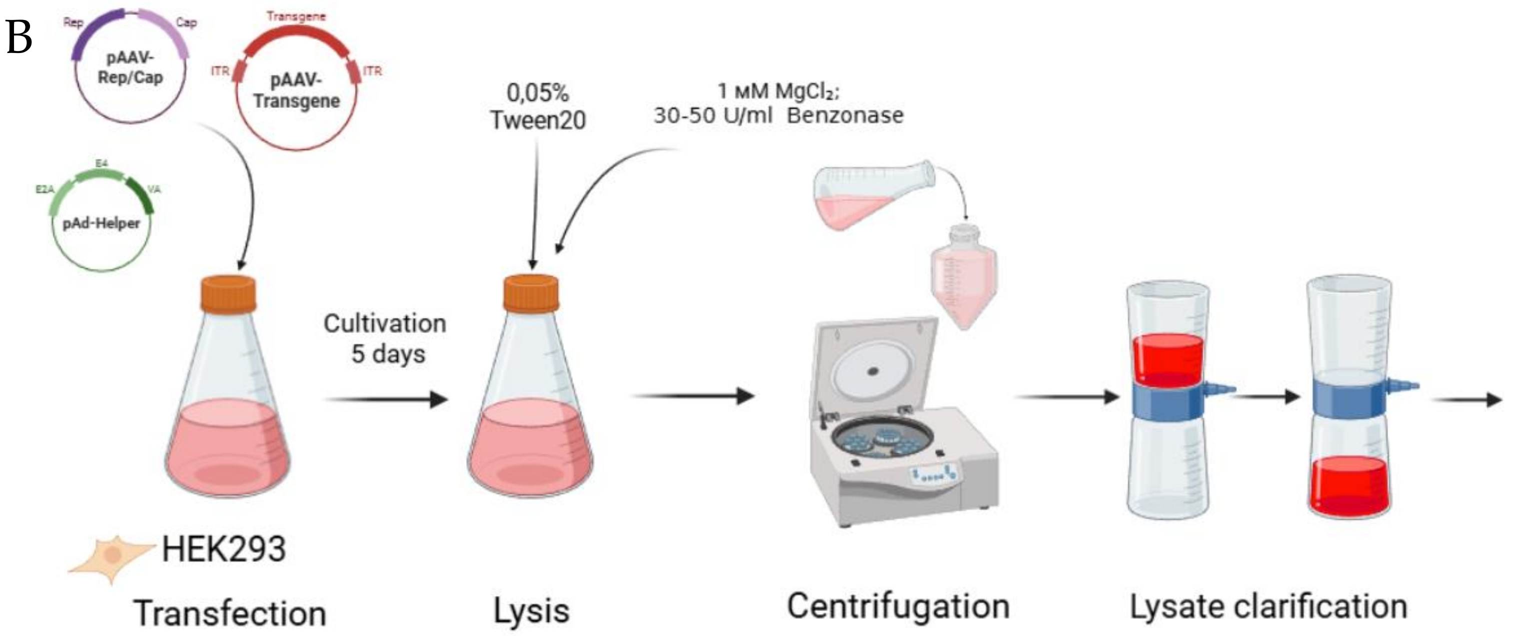

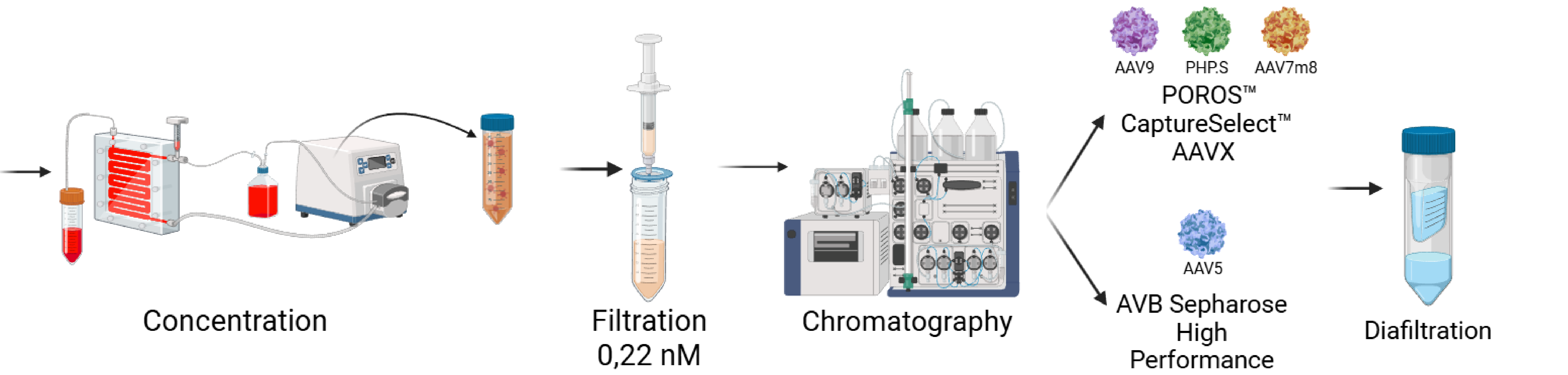

Supplement: Supplementary file 1 [file ijms-27-01366-s001.zip › Figure S4.pdf]
