# Supplementary figures and images for: Functional In Vitro Assessment of rAAV-Delivered Retinol Dehydrogenase 12 (RDH12) Activity
Source: Int J Mol Sci. 2026 Jan 29;27(3):1366. doi: 10.3390/ijms27031366 (PMC12897934; doi:10.3390/ijms27031366)

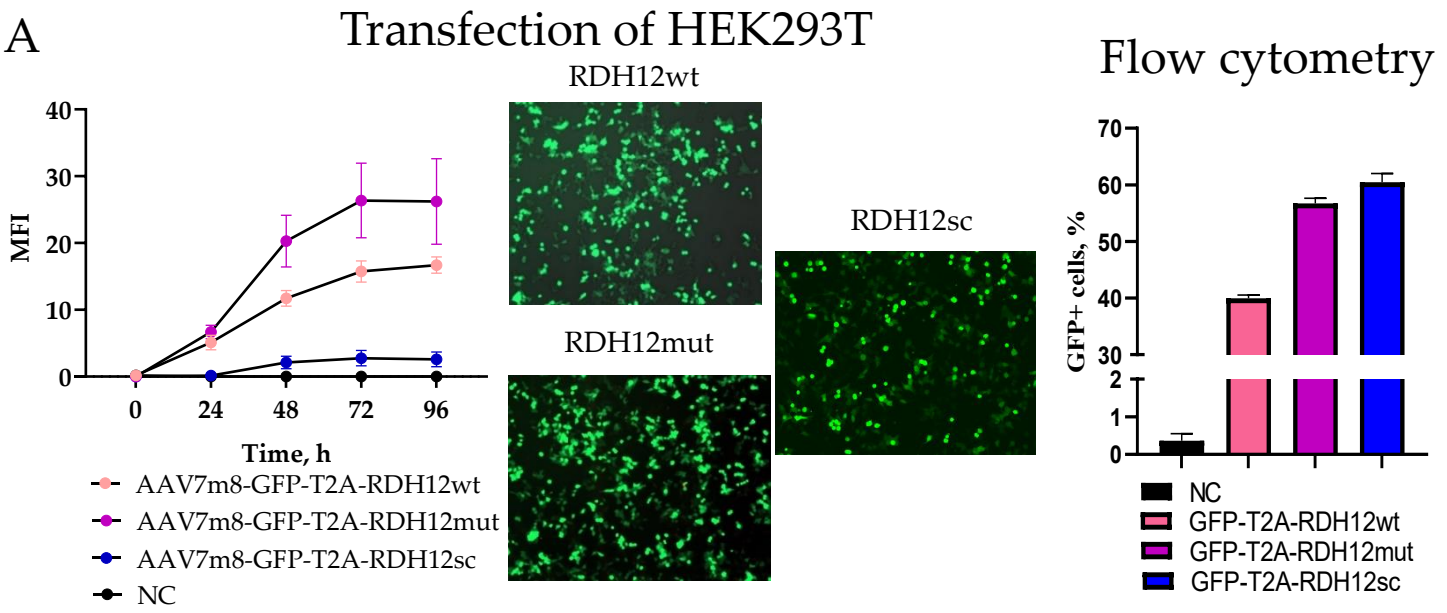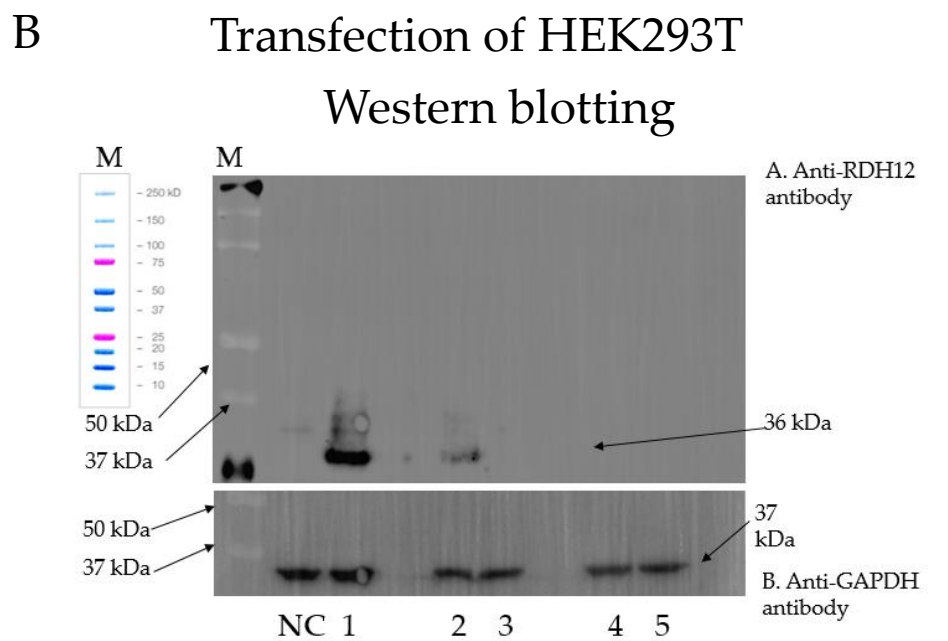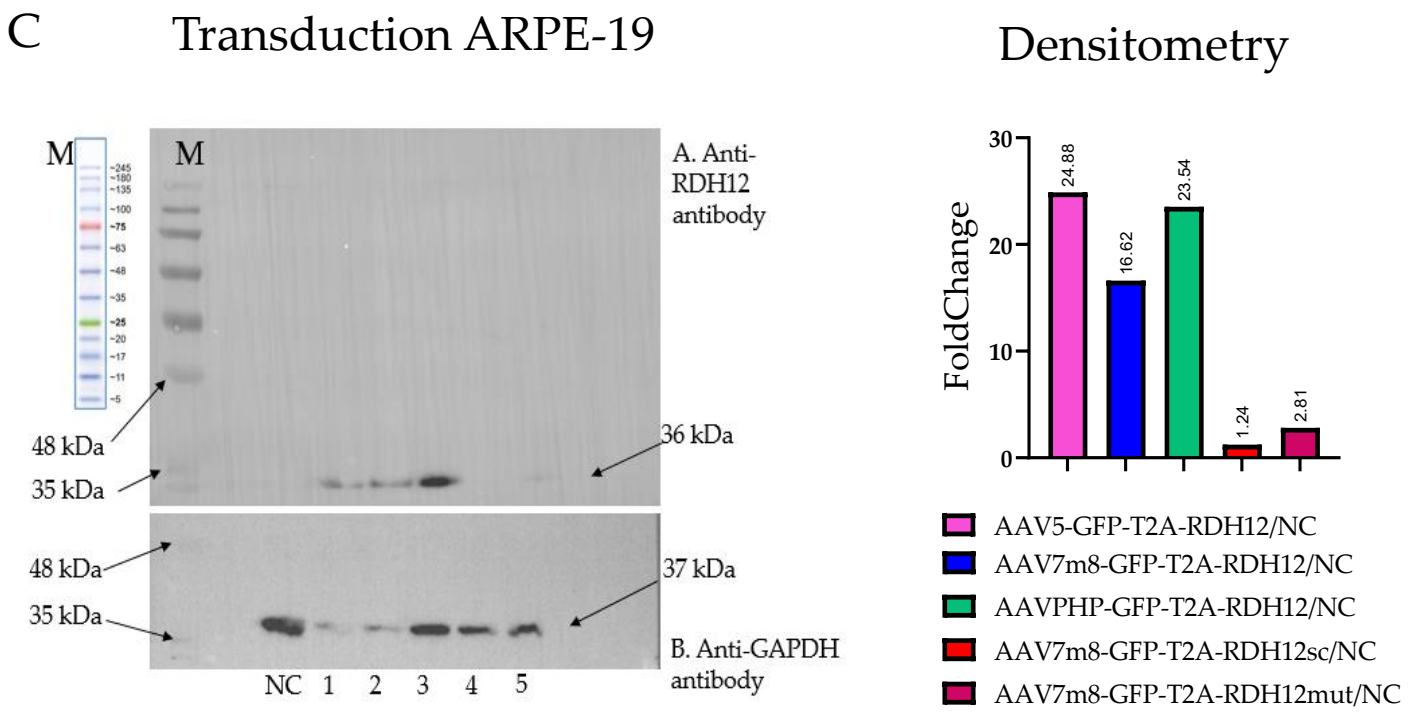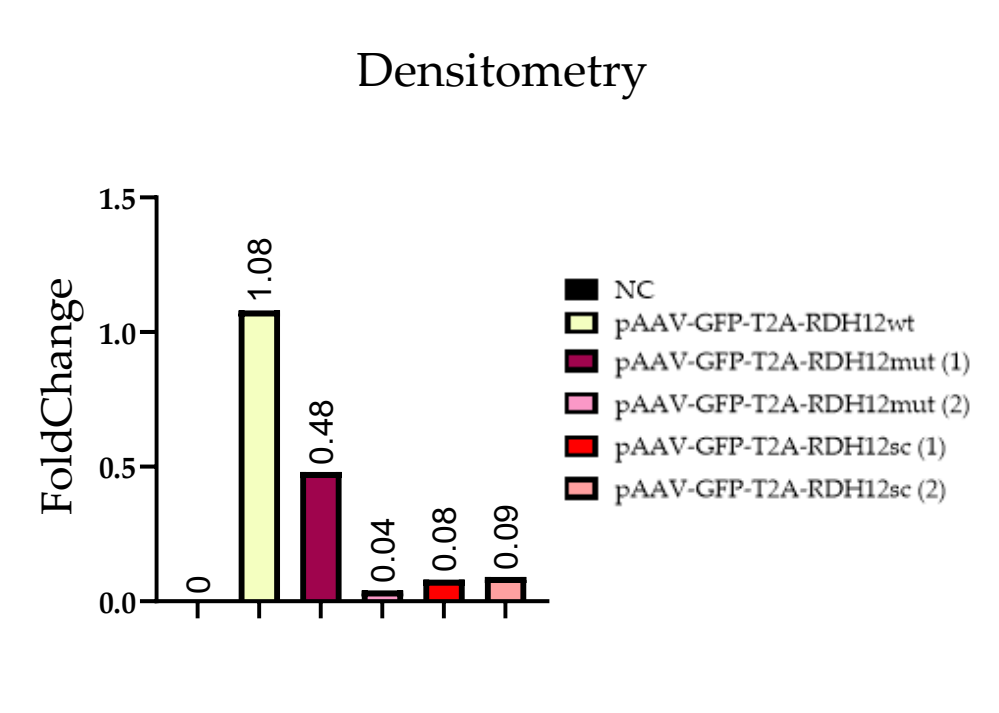

Supplement: Supplementary file 1 [file ijms-27-01366-s001.zip › Figure S1.pdf]

# Gating strategy

A

HEK293

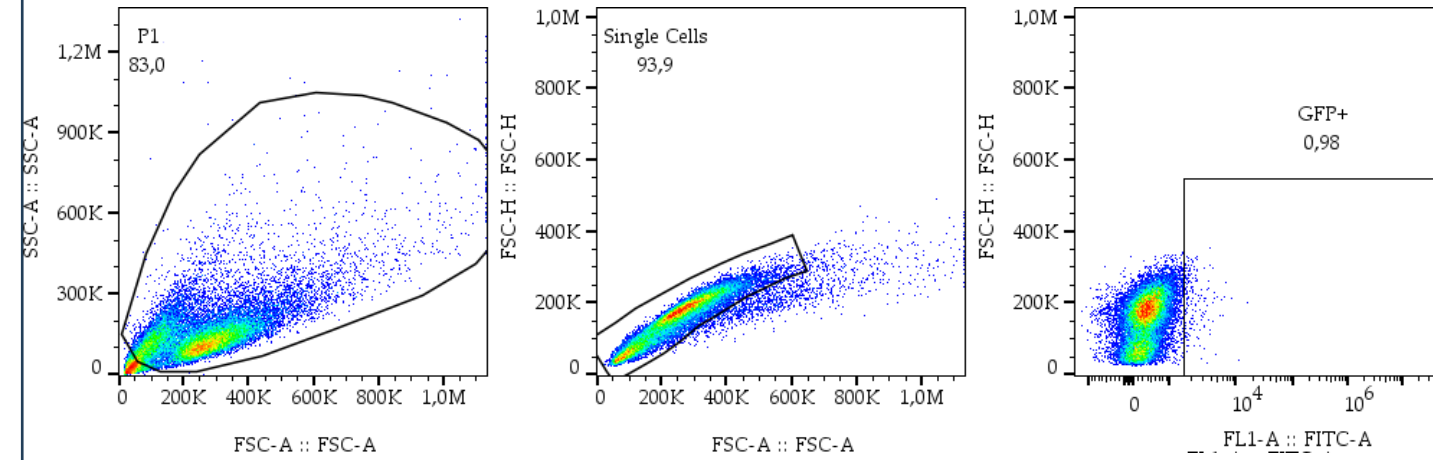

B

ARPE-19

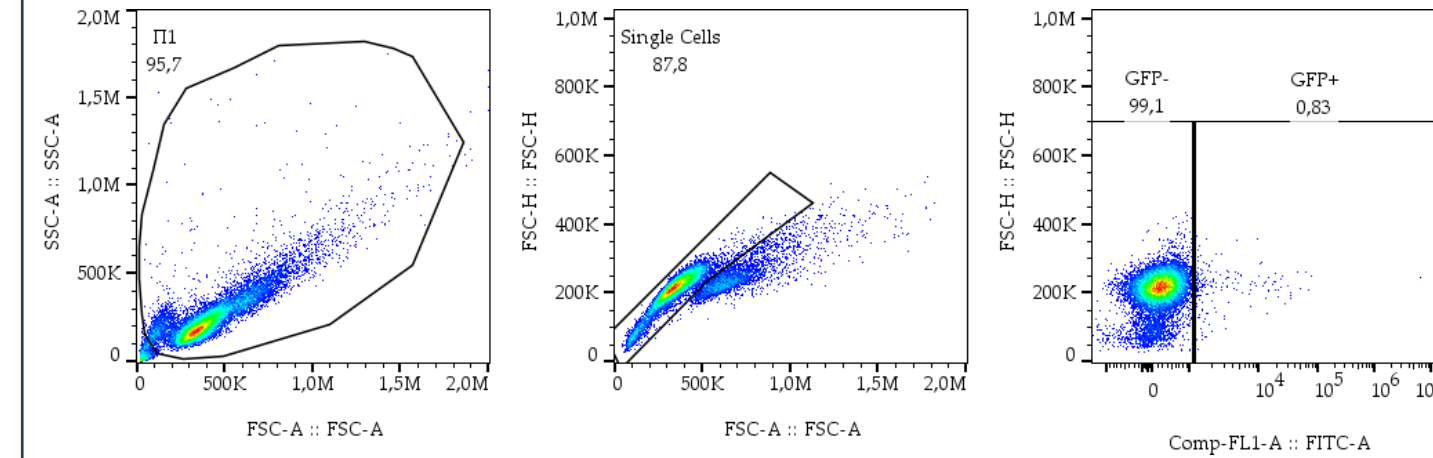

C

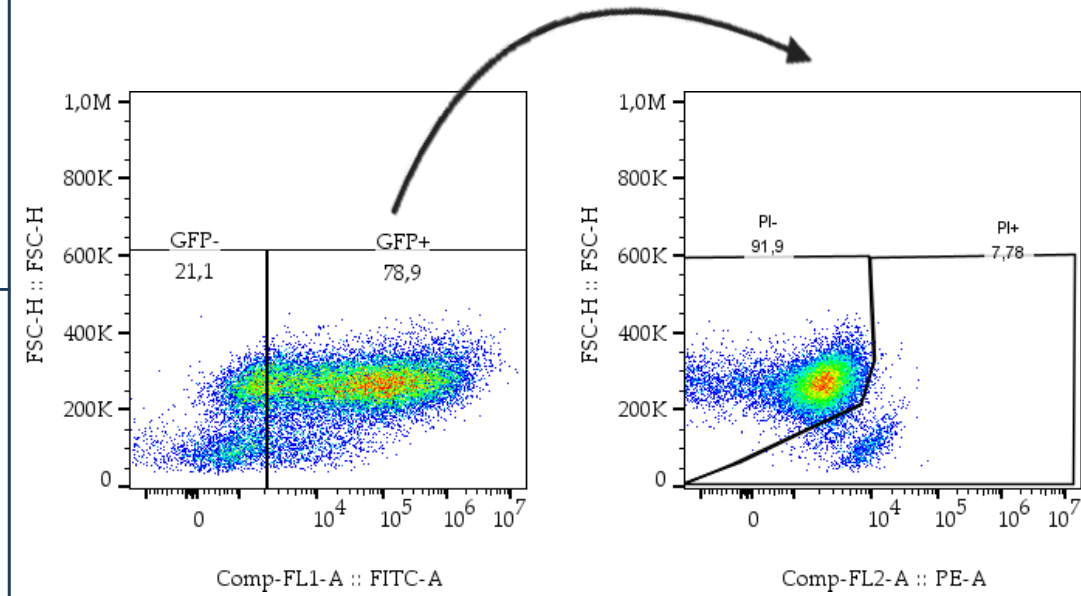

Supplement: Supplementary file 1 [file ijms-27-01366-s001.zip › Figure S3.pdf]
